# Complete Genome Sequence of Clostridium butyricum Strain DKU_butyricum 4-1, Isolated from Infant Feces

**DOI:** 10.1128/MRA.01341-19

**Published:** 2020-03-05

**Authors:** Man-Seok Bang, Hee-Won Jeong, Yea-Jin Lee, Sang-Cheol Lee, Gi Soo Lee, Sungyong Kim, Han-Hwi Lee, Jang-In Shin, Chung-Hun Oh

**Affiliations:** a Department of Medical Laser, Graduate School, Dankook University, Cheonan, Choongnam, Republic of Korea; b Clinical Trial Institute, Dankook University, Cheonan, Choongnam, Republic of Korea; c Department of Oral Physiology, College of Dentistry, Dankook University, Cheonan, Choongnam, Republic of Korea; University of Maryland School of Medicine

## Abstract

Clostridium butyricum is a strictly anaerobic spore-forming bacillus that is commonly present in the gut of humans. We report here the complete genome sequence of Clostridium butyricum strain DKU_butyricum 4-1, isolated from infant feces.

## ANNOUNCEMENT

Clostridium butyricum is a butyric acid-producing Gram-positive anaerobic bacterium that is commonly present as a member of the gut microbiota in humans. This species has been used as a probiotic for the prevention of diarrhea in humans (1). Probiotic bacteria are live microorganisms that give a health benefit to the host. In practice, probiotics can improve the balance of the intestinal microbiota ecosystem of the host, which is beneficial in treating a variety of infant conditions, including infant diarrhea, upset stomach, and eczema, and many health conditions of preterm infants (2, 3). Here, we present the complete annotated genome sequence of Clostridium butyricum strain DKU_butyricum 4-1, isolated from infant feces.

Fecal samples were prepared as previously described (4). Briefly, fecal samples were collected from breastfed infants less than 3 months old at a hospital and a nearby postpartum care center in Cheonan-Asan, Republic of Korea, between 2012 and 2013. For the isolation of Clostridium butyricum from infant feces, collected fecal samples were incubated at 30°C in an anaerobic chamber (Forma Anaerobic System; Thermo Fisher Scientific, USA) using a gas phase of N_2_/H_2_/CO_2_ (80%:10%:10% [vol/vol/vol]) for 3 days. After enrichment, they were further serially diluted and plated on reinforced clostridial medium (RCM; BD) for anaerobes in an anaerobic chamber at 30°C for 48 h. The colonies were purified by repeated streaking onto the agar medium and were subcultured periodically. In total, 102 isolates showing a bubble with soap skin were selected as the candidates, and their biochemical profiles were confirmed using the API 20A system (bioMérieux, UK) in order to identify the isolates. One strain, DKU_butyricum 4-1, which showed the active formation of gas bubbles, was chosen for further study, and its identification was confirmed by whole-genome sequencing.

DNA was prepared using the Wizard genomic DNA purification kit (Promega) following the manufacturer’s instructions. Whole-genome sequencing of Clostridium butyricum strain DKU_butyricum 4-1 was performed with a 20-kb SMRTbell library (PacBio DNA/polymerase binding kit P6) on the RS II sequencing platform (Pacific Biosciences, USA) using C4 chemistry with 8 single-molecule real-time (SMRT) cells at Macrogen (Seoul, Republic of Korea) (5). In total, 147,028 PacBio subreads (average subread length, 8,579 bp; subread *N*_50_, 11,854 bp) of *C. butyricum* strain DKU_butyricum 4-1 were generated.

The raw PacBio reads were corrected, trimmed, and *de novo* assembled using the Canu version 1.7 protocol (6). The result of the assembly was two contigs with an *N*_50_ value of 3,867,296 bp. Based on the assembly information, this genome consists of 4,636,588 bp divided into 1 closed circular chromosome of 3,867,296 bp (G+C content, 28.8%; coverage, 210×; GenBank accession number CP039705) and 1 circular plasmid of 769,292 bp (G+C content, 28.3%; coverage, 186×; accession number CP039706).

The genomes were annotated with Prokka version 1.12b software (7). There are 3,593 genes, 3,468 coding DNA sequences (CDSs), 36 rRNAs, 88 tRNAs, and 1 transfer-messenger mRNA (tmRNA) on the chromosome and 679 genes and 679 coding sequences (CDSs) on the plasmid.

### Data availability.

The complete genome and plasmid sequences of *C. butyricum* strain DKU_butyricum 4-1 were deposited in GenBank under accession numbers CP039705 and CP039706, respectively. The associated BioProject, BioSample, and SRA accession numbers are PRJNA534376, SAMN11489284, and SRR10344400, respectively.
